# Revising the Tool for Assessing Cultural Competence Training (TACCT) for curriculum evaluation: Findings derived from seven US schools and expert consensus

**DOI:** 10.3885/meo.2008.Res00272

**Published:** 2008-07-30

**Authors:** Désirée A. Lie, John Boker, Sonia Crandall, Christopher N. DeGannes, Donna Elliott, Paula Henderson, Cheryl Kodjo, Lynn Seng

**Affiliations:** * University of California, Irvine School of Medicine, Orange, California, USA; † Wake Forest University School of Medicine, Winston-Salem, North Carolina, USA; ‡ Howard University College of Medicine, Washington, DC, USA; § Keck School of Medicine at the University of Southern California, Los Angeles, California, USA; ‖ David Geffen School of Medicine at the University of California, Los Angeles, Los Angeles, California, USA; ¶ University of Rochester School of Medicine, Rochester, New York, USA; ** University of Pennsylvania School of Medicine, Philadelphia, Pennsylvania, USA

**Keywords:** cultural competence, curriculum tool, evaluation, validation

## Abstract

**Background:**

The 67-item TACCT currently used for needs assessment has potential for evaluating evolving cultural competence (CC) curricula.

**Purpose:**

To validate a shortened, more practical TACCT measure.

**Methods:**

The 67-item TACCT was administered to students and course directors at US schools. Course directors and students reported which of 67 TACCT items were taught. Intraclass correlation coefficients (ICC) examined faculty-student agreement. Under-addressed content was identified. A new and shortened TACCT configuration was proposed and validated with expert educator input.

**Results:**

Across-school faculty and student response rates ranged from 75% to 100%. Aggregate ICC was 0.90 (95% CI: 0.84, 0.94) for the 67-item TACCT, demonstrating faculty-student agreement. Experts agreed on reduction from 67 to 42 items and domain revision from five to six domains to match under-addressed content. Item analysis showed high internal consistency for all 6 new domains and the total revised 42-item TACCT.

**Conclusions:**

A shorter, more practical TACCT measure is valid and reliable and focuses on under-addressed CC content. Use for curricular evaluation is suggested.

There is growing evidence that improving cross-cultural communication skills of healthcare providers is associated with better patient outcomes.^1^–^3^ Examples of cultural competence (CC) curricula are available, but evidence-based tools for evaluating the impact of CC curricula are needed. Published US recommendations for cultural competence training provide guidelines.^4^–^7^ However, a recent systematic review examining the robustness of cultural competence education evaluations concluded that the lack of methodological rigor limited the value and impact of studies reporting the effectiveness of specific educational interventions and asserted that attention should be paid to the proper design, evaluation, and reporting of such training programs and courses.^8^ Another systematic review examined a wide array of tools for assessing learner attitudes and CC curricula and noted little standardization for use across medical schools.^9^ For example, a survey of 19 US medical schools in 2001^10^ identified 8 important content areas in cross-cultural education for medical students and suggested a standard nomenclature for measuring ‘the success of cross-cultural education curricula’. In this paper we focus on the need to identify core content that addresses knowledge, skills and attitudes leading to cultural proficiency and competence promoting improved healthcare outcomes in the context of medical encounters with diverse patients. The primary term we choose to use for this purpose is ‘cultural competence’ to broadly cover an array of terms used in the literature ranging from ‘cultural humility’ to ‘diversity’ to ‘cross-cultural communication skills’.

The accreditation standards of the Liaison Committee on Medical Education (LCME)^11^ specify two cultural competence guidelines. First, ‘Medical students should learn to recognize and appropriately address gender and cultural biases in health care delivery, while considering first the health of the patient.’ Second, ‘The faculty and students *must* demonstrate an understanding of the manner in which people of diverse cultures and belief systems perceive health and illness and respond to various symptoms, diseases, and treatments.’ Based on the LCME guidelines, the Association of American Medical Colleges (AAMC) developed the TACCT for use as a needs assessment tool. The TACCT was designed by a consensus panel of experts. Its intended use was to measure the degree to which the various content elements of CC occur throughout the curricula of US and Canadian medical schools from the perspective of teaching faculty. The measure has five domains comprising of 67 CC content-specific items (or learning objectives) representing knowledge, skills, and attitudes, mirroring a prior AAMC curriculum assessment measure for palliative care.^14^

Since 2004, the National Institutes of Health (NIH) has funded 18 US medical schools to design, implement, and disseminate model CC curricula.^15^ Separately, in 2005 the AAMC also supported four California medical schools to develop and implement model CC curricula. Two schools received both awards, for a total of 20 schools in the 2 consortia. Investigators at one funded school, the University of California, Irvine (UCI), initially administered the TACCT to both faculty and students and found high congruence (intraclass correlation coefficient =0.89) between faculty and student perceptions of whether CC content, as expressed in the TACCT items, was presented in the extant curriculum. Furthermore, students were significantly more likely to identify content as being covered compared to course directors overall.^16^ Faculty and students also agreed on content least likely to be addressed which fell into three broad content areas: health disparities, bias and stereotyping, and community strategies. Subsequently, the TACCT was used by six additional schools among the NIH and AAMC awardees to conduct a baseline curriculum needs assessment.

However, in its original form the TACCT poses a number of challenges for routine use (e.g., annual or repeated administration) in curriculum evaluation. Mainly, the number of items or learning objectives (n = 67) is daunting at first glance to potential respondents. Also, the wording of some items appears to overlap and requires respondents to make fine discriminations in intended meaning (e.g., ‘identify physician biases that affect clinical care,’ ‘value the need to address personal bias,’ ‘recognize how physician biases impact care’). Furthermore, distinctions between generic communication objectives (e.g., ‘value curiosity, empathy and respect’) and CC-specific communication objectives (e.g., ‘respect patient's cultural beliefs’) are not clearly made. Finally, the professional behavior of self-reflection in relation to CC is not explicitly included but is instead embedded within other domains.

## Purpose

Our objectives were, first, to identify areas of least-addressed content and second, to apply expert judgment and statistical principles to develop a shorter TACCT.

## Methods

**Study Sample** - Seven US schools divided between the east and west coast regions (three and four schools, respectively) participated in the survey; three were state institutions. The self-selected schools belonged to two consortia of collaborative institutions awarded grants to implement cultural competency curricula by the NIH (six schools) and the AAMC (two schools). One school belonged to both groups, for a total of 7 participating schools. The respective institutional review boards at the seven schools approved the study. Respondents were both medical students in the clinical phase of training (third year medical students [MS3] or fourth year medical students [MS4], n = 662), who had completed at least the required core curriculum, and course and clerkship directors (n = 144) of the core required medical school courses at the respective institutions. Reported institutional data are de-identified.

**TACCT Administration and Data Collection** - Five schools (schools 1, 3, 5, 6 and 7) administered the 67-item TACCT^12^ to students as a written questionnaire either during a Clinical Practice Examination or during a required class. Two schools (schools 2 and 4) first administered the TACCT to students online using email and a web link. A subsequent face-to-face administration was used with non-responders. Faculty respondents were surveyed by email listserv solicitation followed by a face-to-face administration at school 1 and by written questionnaire only at the other schools. The method of administration (online vs. face-to-face pen and pencil) was determined by each school based on past experience with survey response rates for their particular respondents, with the purpose of maximizing response rate.

Students and faculty were instructed to check all items (scored as ‘yes’ = 1) that they felt had been adequately addressed in the curriculum medical students (MS) or in their own courses (faculty). Items unchecked were scored as ‘not addressed’ = 0. Detailed explanation of individual TACCT item content was not provided. Respondents were asked to interpret each item at face value as they understood it and to not check items they did not understand or that were not taught in the required curriculum.

TACCT administration was completed within a 12-week timeframe by both students and faculty in all schools. Average time for completion of the TACCT was 15 to 20 minutes.

**Data Analysis** - The frequency of ‘yes’ responses to each TACCT item was tabulated separately for students and faculty. Mean percentage item scores were computed (sum of ‘yes’ responses divided by number of respondents). Domain scores were computed from summing items that belonged to each of the five conceptual TACCT domains. Finally, within each domain, knowledge, skill, and attitude scale scores were computed from summing the pertinent items belonging to each respective category. The authors also examined the distributions of faculty and student TACCT item scores to identify clusters of items denoting content areas defined as ‘not adequately addressed’ in the curriculum (i.e., in the lowest quartile of responses). The intraclass correlation coefficient (ICC) was calculated to evaluate the degree of concordance among faculty and student responses.

Because of concerns about faculty and student responses being skewed in opposite directions, nonparametric Exact Mann-Whitney Tests compared faculty and student responses on each TACCT item and with the corresponding domain and scale scores. One-way analysis of variance (ANOVA) examined potential between-school differences on both domain and scale scores. Significant *F*-tests were followed by pair-wise mean comparisons by either Neuman-Keuls (homogenous group variances) or Games-Howell tests (heterogeneous variances). The researchers used the nominal, two-sided α < 0.05 for testing statistical significance. Because we computed multiple comparisons, the conservative Bonferroni correction (i.e., α divided by number of contrasts) was applied to each family of comparisons (e.g., student versus faculty within each school). All analyses were performed using SPSS 14.0 for Windows (SPSS Inc., Chicago, Illinois).

**TACCT Review Process** - The expert survey occurred within eight months of completing the 7-school survey. Over a three-month period, experts defined as educators with experience administering and using the TACCT were identified and recruited from among the NIH Consortium and the AAMC awardees. They first were asked (by telephone, email, and in person) if they had used the TACCT for educational needs assessment at their institutions. They then received the 67-item TACCT and findings from both the prior UC-Irvine study^16^ and the recently completed 7-school study. A diversity education representative from the AAMC, who was involved in the construction and dissemination of the TACCT, was included at this stage of the review process. The experts were asked to independently (a) review the 67-item TACT, (b) suggest alternative clusters of items and reconfigured domains for curriculum evaluation based on either the studies’ findings or their own experiences with the TACCT, and (c) add their individual suggestions, explanations and comments about revising and improving the measure. Based on the composite suggestions, the TACT was restructured and then sent back to the experts for further review and to achieve consensus agreement about changes made to the measure. At a joint meeting of both the NIH Consortium and AAMC awardees convened by the AAMC Cultural Competency Education committee in September 2007 and attended by all 20 schools’ representatives, the restructured TACCT was subjected to final peer review and scrutiny.

**Reliability Analysis of Restructured TACCT** - Using the survey responses from the seven schools, item analysis was performed using conventional methods. That is, internal consistency reliabilities were assessed by computing Cronbach's coefficient alpha (α) for the set of pertinent items comprising each newly configured domains. Within each domain, we computed the point-biserial correlation coefficients of each individual item score with the total domain score in which the items clustered. Also, for within-domain analyses, α was recomputed by deleting each item score from the total domain score to assess the relative contribution of each individual item to its respective broader domain score.

## Results

**Response Rates and Student Demographics** - Student response rates varied from 75% to 90%; faculty rates ranged from 95% to 100%. Ethnic/racial profiles of students represented significant diversity among the student body within each school (Table 1). Ranges within schools included 6% to 75% non-Hispanic white, 2% to 75% African-American, 1% to 28% Hispanic/Mexican-American, and 4% to 40% Asian-American. Percentage of males ranged from 45% to 55%. The IRB status of the research protocol precluded identifiers to correlate individual responses with respondent.

**Table 1:** Responding Student Demographics for 7 US Schools: Self-identified respondent ethnicity for school overall (not respondent demographics)

Characteristics	Total n (%)						
**School**	1	2	3	4	5	6	7
**Class of (year)**	2007	2006	2006	2007	2006	2006	2006
	(MS3)	(MS4)	(MS4)	(MS3)	(MS4)	(MS4)	(MS4)
**Ethnicity**							
White	49(53.3)	6 (5.8)	64 (40.0)	19(19.2)	77 (59.7)	74 (74.7)	61 (62.2)
African American	2 (2.2)	77 (74.8)	6 (3.8)	27 (27.3)	12 (9.4)	9(9.1)	19(15.3)
Hispanic/Mexican American	10(10.9)	1 (1.0)	13 (8.1)	28 (28.3)	10.7(10.7)	4 (4.0)	4(4.1)
Asian	21 (22.8)	4(3.9)	64 (40.0)	23 (23.2)	23 (17.6)	12(12.1)	14 (12.2)
Native American	8 (8.7)	0	2(1.3)	1(1.0)	0	0	0(6.1)
Other/no response	2 (2.2)	12(11.7)	11(6.9)	1(1.0)	3 (2.5)	0	0

**Total**	92	103	160	99	129	99	98

LEGEND

MS = Medical Student, Year = Year of graduation

**Item-Level Findings** - Students consistently responded ‘yes’ at a higher rate than faculty to every one of the 67 TACCT items (Table 2, Appendix I). Using a conservative criterion for statistical significance (*p*<.001), students provided a statistically higher mean percentage of ‘yes’ responses to individual TACCT items on 47% of items within schools (range 3% to 90%) and on 100% of items in the aggregate seven schools. Although students responded affirmatively more often than faculty, the two groups generally agreed about what specific elements of cultural competence instruction did or did not occur in the first three years of the curriculum. That is, students and faculty rank ordered in similar fashion the relative occurrence (or non-occurrence) of the cultural competence content represented in the 67 separate TACCT items (Table 2). The range of intraclass correlation coefficients (ICC) within individual schools was 0.70 to 0.89. For the aggregate seven schools the ICC was 0.90 (95% CI: 0.84, 0.94) (Table 3). Three were no statistically significant differences by pattern of student demographics in the level of concordance seen between faculty and student respondents (data not shown).

**Table 3:** Faculty-Student Agreement about Content Coverage of 67 TACCT Items

Measured by Intraclass Correlation Coefficients (ICC), with 95% Confidence Intervals) for Seven Schools								

School Number								
	1	2	3	4	5	6	7	All
ICC	0.89	0.77	0.73	0.88	0.70	0.70	0.85	0.90
95% CI	0.82–0.93	0.63–0.86	0.57–0.84	0.80–.093	0.51–.081	0.51–0.82	0.75–0.90	0.84–0.94

**Domain and Scale-Level Findings (67-item TACCT)** - Examination of the possible domain and scale scores produced a similar pattern to that found with individual TACCT items (Table 4). Students, compared to faculty, yielded statistically significantly higher mean scores on 71% (range 4% to 100%) of domain and scale scores within schools and 100% of the time in the aggregate. Schools 2, 3 and 5 were notable in their magnitudes of differences between mean total TACCT scores from faculty and students (44%, 42%, and 47% respectively). The smallest faculty-student TACCT total score difference was 20% in School 4 (Table 4). Considering only students’ responses, significant (*p*<.0005) between-school differences occurred on every domain and scale score, except for Domain V Knowledge (‘Cross Cultural Clinical Skills’), where all schools produced similar mean scores (78%, range 69% – 84%). In contrast, no significant between-school differences emerged from analysis of faculty domain and scale scores. Students from Schools 2, 3, and 6 consistently responded ‘yes’ at rates greater than 80% to TACCT items categorized as knowledge, skill, and attitude. Consequently, these three schools had significantly higher total TACCT scale scores than their four counterparts (Table 4).

**Table 4:** Domain Mean Frequency of 'Yes' (Percentage) Responses by Domain (for 67-item TACCT) by Medical Students and Faculty Course Directors at Seven US Schools

School	1	1	2	2	3	3	4	4	5	5	6	6	7	7
Respondents*	S	F	S	F	S	F	S	F	S	F	S	F	S	F
Sample Size (n)	n = 69	n = 25	n = 92	n = 24	n = 148	n = 19	n = 66	n = 18	n = 129	n = 26	n = 76	n = 11	n = 82	n = 22

Domain I: Rationale, Context, & Definition	72	33	83	41	83	47	63	46	75	33	78	62	61	24
Domain II Key Aspects of Cultural Competence	72	39	83	51	82	51	70	56	71	30	81	68	67	33
Domain III: Impact of Stereotyping On Medical Decision-Making	53	30	59	21	65	27	52	33	54	11	62	42	51	18
Domain IV: Health Disparities and Factors Influencing Health	57	28	80	33	75	28	54	27	65	19	75	45	56	18
Domain V: Cross Cultural Clinical Skills	73	41	85	30	88	42	71	53	76	22	80	61	73	26
Total Scores	**68**	**36**	**80**	**36**	**82**	**40**	**65**	**45**	**70**	**23**	**78**	**57**	**64**	**25**
(SD)	(25)	(28)	(34)	(19)	(26)	(18)	(23)	(23)	(24)	(22)	(25)	(27)	(27)	(20)

*S = Students F = Faculty

**Under-Addressed Content Areas** - Among the seven schools, using the 67-item TACCT, we identified 19 TACCT items for students and faculty that fell within the lowest quartile of ‘yes’ responses and portrayed, by definition, under-covered curriculum content (see Table 2 ranking and shaded area). Among this item set, 14 were identical (Table 2, shaded and in bold) for the two groups (students and faculty) and clustered into three broad content areas. The first broad content area was Community Strategies. It was represented by the following TACCT items: ‘describe community-based elements’, ‘describe methods to identify community leaders’, ‘propose a community-based health intervention’, ‘describe community partnering strategies’ and ‘collaborate with communities to address needs’. The second broad content area was Health Disparities. This area was framed by items that include: ‘critically appraise literature on health disparities’, ‘gather and use data as in HP (Healthy People) 2010’, ‘describe social cognitive factors’, and ‘concretize the epidemiology of disparities’. The third under-addressed content area was labeled Bias/Stereotyping and contained the following cluster of items: ‘show strategies to reduce bias in others’, ‘value the historical impact of racism’, ‘describe historical models of health beliefs’, and ‘strategize ways to counteract bias’.

**Expert Consensus Results** - Nine experts and the AAMC representative returned suggestions for revising the TACCT. They agreed that the under-addressed curricular areas (Health Disparities, Community Strategies and Bias/Stereotyping) should each occupy a separate domain for ongoing curriculum evaluation to ensure that they were distinctly tracked as new CC curricula were introduced. They also agreed that individual TACCT items (or objectives) should be preserved in their original form of wording and not reworded. Thus, no new TACCT items were introduced in the restructuring process. All 9 experts agreed that there was over-representation of objectives addressing bias and stereotyping in the original TACCT and suggested reducing the number of knowledge, attitude, and skill items in the bias/stereotyping content from 17 items in the 67-item TACCT to 6 items (see Table 5, Appendix II, Domain II). The experts agreed that distinct domains representing cross-cultural communication skills, interpreter use skills, and self-reflection in the context of the culture of medicine should be included (see Table 3, Domains IV, V and VI). Removing redundant objectives from the original TACCT in total reduced the measure by 25 items, arriving at a final number of 42 items (or objectives). The final re-structured TACCT (Table 5 Appendex II) comprised six renamed domains, each with no more than 10 objectives: health disparities, community strategies bias/stereotyping, cross-cultural communication skills, use of interpreters and self-reflection/culture of medicine. The revised 42-item measure was sent back to the experts for review with full consensus reached on the new domains and domain items. At a meeting of both consortia in September 2007, the 42-item TACCT was reviewed by representatives of 20 schools and no further revisions were advocated by the group.

**Item Analysis of Restructured TACCT** - Using the 7-school data on the 67-item TACCT, the inter-rater agreement as measured by ICC between medical student and faculty responses to the 42 items of the reconfigured TACCT was .905 (95% CI, .816, .947). Reducing the number of objectives from 67 to 42 thus did not affect medical student-faculty agreement. Conventional item analysis including Cronbach's coefficient alpha showed solid results in support of the restructuring of knowledge, skill and attitude domains in the newly configured TACCT. Cronbach's α for each of the six new domains ranged from .803 to .875. Overall, the α coefficients for all reconfigured knowledge, skill, and attitude objectives were .914, .923, and .857, respectively. The total new TACCT with 42 items had α=.964 (see Table 5). When α was recalculated by deleting each constituent item score from the new domain scores to which they clustered, the resulting coefficient always was lower then when the item was included, suggesting that each item made a positive contribution to the variance in the total domain score (data not shown). The item score-total score correlation coefficients in content and knowledge, skill and attitude domains were consistently moderate to high by conventional definition (i.e., never<.40).

## Discussion and Conclusions

The present study with seven geographically dispersed schools replicated and further demonstrated the reliability and concordance of student and faculty responses shown in a prior single-school study. Likewise, three under-addressed content areas identified in the single-school study were confirmed by administering the original 67-item, 5-domain TACCT. An additional study arm using expert review and consensus yielded a restructured TACCT that ostensibly improved its utility for curriculum evaluation. Finally, examination of the psychometric characteristics of the restructured 42-item TACCT showed that reducing the length of the measure did not detract from its internal structure and reliability.

Since students responded according to their instructional exposure across at least three years of the curriculum, whereas faculty course directors responded in the more limited context of instruction provided only within their own formal courses, it perhaps was not surprising that students systematically checked more TACCT items. One reason for these differences may be that students included experiences with cultural competence content in the ‘informal or hidden curriculum’ in responding. Concomitantly, observed variability in schools may reflect a combination of real differences in the informal and formal curricula and perhaps differential recall of both experiences. Content least likely to be addressed was similar for each school and overall. The three content areas found least likely to be addressed in all schools were Community Strategies, Health Disparities, and Bias/Stereotyping. This remarkable symmetry underscores the importance of developing curriculum that can be used across schools to address these content areas.

It was reassuring that restructuring the TACCT into six renamed domains that included the least addressed areas identified in the 7-school study with a reduction by 25 items (or objectives) did not reduce the internal consistency reliability of the TACCT overall, each domain, and the separate components of knowledge, skill and attitude. The total new TACCT Cronbach's alpha and individual alphas for the new domains support the future use of the restructured 42-item TACCT as a curriculum evaluation measure. The introduction of a new domain (VI) of ‘self-reflection, culture of medicine’ echoes the *a priori e*xpert judgment solicited from educators and this judgment was affirmed by the high internal consistency reliability (Cronbach's α=.803) of this 5-item domain.

Individual schools have different curricular orientations, local community needs, and diverse geographic, cultural, and ethnic backgrounds among students and faculty. There may be differences with respect to the role and importance of cultural competence education for health professionals and with respect to the manner in which this education should be offered. By providing a revised TACCT based on results obtained from multiple schools, our study contributes to the need to address measuring the effectiveness of curricular change in CC education. The restructured 42-item TACCT is more practical and user-friendly than the 67-item TACCT and specifically recognizes key areas of curricular content currently under-addressed in most schools. As such, it is a feasible alternative to the longer original 67-item TACCT for schools undergoing curricular change in CC instruction.

The strengths of the current study are that the seven schools were diverse in respective student demographics, but the TACCT administration method was relatively uniform across the schools, data collection was conducted within a short timeframe, and there were high response rates from both students and faculty. The use of peer review by experts from two consortia representing 20 US schools addressing CC education as a common goal is another strength. Limitations of the study are both the relatively small number of schools represented and potential variability in interpretations of individual TACCT items by respondents within and across schools. The current study does not address the content of the informal curriculum that student responses may have included, and we believe that this aspect of the CC curriculum may best be addressed in greater depth using qualitative methods. This current study was not intended to prescribe particular curricula to address the efficacy of teaching in particular content areas such as community strategies, health disparities or specific cross-cultural communication skills, as such curricula are well described and available in the literature.^20^–^25^ The applicability of the TACCT to non-US and non-Canadian schools not guided by LCME standards is uncertain. Despite the shortcomings described, concordance among faculty and students in our multi-school needs assessment was remarkably high and consistent within each school. In addition, agreement among the peer experts familiar with CC curricula and the TACCT was consistently high, and their findings were in turn supported by the feedback from experts from all 20 NIH and AAMC funded schools. Further testing and use to examine the validity of the restructured 42-item TACCT as a curriculum evaluation measure is needed.

In summary, the TACCT in its restructured format is practical, has face and content validity, and is a reliable instrument to administer with straightforward instructions to medical students and faculty. However, despite its comprehensive coverage of CC learning objectives, the original or revised TACCT is not intended to be prescriptive, in that it does not identify specific or best teaching for meeting the objectives or for evaluating learners. That particular task of curriculum implementation, in our opinion, is best achieved at each school for its curriculum by its own educators, because, like other curricular areas, there is not a ‘one size fits all’ approach for CC education as the literature reporting myriad CC curricula suggests. Students may have greater exposure to cultural competence in the entire formal and informal curriculum experience, in comparison to faculty whose contact is inherently limited to parts of the formal curriculum. Thus, we advocate that both student and faculty viewpoints should be considered in planning CC curricula. Furthermore, the diversity of the population residing in the geographic location surrounding the institution may also influence responses to the TACCT. Future studies should attempt to separate the latter effects from that of the medical school curriculum. Combining an externally validated objective measure of curricular coverage in cultural competence for each school (such as an external review of syllabus and teaching materials and in-depth interview of students and course directors or focus group studies, for example) with the current results may allow confirmation of this observation and help distinguish the informal from the formal curriculum.

We recommend that the restructured 42-item TACCT be used both for baseline needs assessment and to evaluate the impact of introducing new CC education, especially when repeated (i.e., pre- and post-training) administrations are contemplated.

## Appendix I

**Table 2:** Frequency Differences (Confidence Intervals) and Rank Order of Faculty and Student ‘Yes’ responses of all 67 TACCT items

		Medical Students (N = 662)		Faculty (N = 144)	
			
TAACT Inventory Item Descriptions	95% C.I.: MS-Faculty Mean Difference	TAACT Inventory Items (Highest-Lowest)	Mean% (SD)	TAACT Inventory Items (Highest-Lowest)	Mean% (SD)
DI. CULTURAL COMPETENCE RATIONALE, CONTEXT, AND DEFINITION					
K1. Define race, ethnicity, and culture	.464–.612	DII A4	.91 (.292)	DII A4	.72 (.449)
K2. Identify how race and culture relate to health	.221–.344	DI K2	.90 (.300)	DIV K1	.63 (.484)
K3. Identify patterns of national data on disparities	.297–.458	DII A2	.89 (.310)	DI K2	.62 (.488)
K4. Describe national health data	.210–.381	DI A2	.88 (.326)	DI A2	.59 (.493)
S1. Discuss race & culture in the medical interview	.281–.441	DII K2	.88 (.326)	DII A2	.57 (.497)
S2. Use physician assessment tools	.340–.509	DIV K1	.88 (.236)	DII K2	.53 (.501)
S3. Concretize epidemiology of disparities	.252–.427	DV A1	.88 (.330)	DII K4	.53 (.501)
A1. Describe own cultural background and biases	.390–.549	DII K3	.87 (.335)	DV S1	.53 (.501)
A2. Value link between communication & care	.224–354	DIII K3	.87 (.340)	DV A1	.53 (.501)
A3. Value importance of diversity in healthcare	.234–.380	DIV K2	.85 (.358)	DI A3	.52 (.501)
DII. KEY ASPECTS OF CULTURAL COMPETENCE					
K1. Describe historical models of health beliefs	.252–.426	DIII K2	.85 (.361)	DII K5	.51 (.502)
K2. Recognize patients’ healing traditions & beliefs	.279–.410	DV S1	.84 (.371)	DII S2	.51 (.502)
K3. Describe challenges in cross-cultural community	.347–.480	DIII A2	.83 (.374)	DIV K2	.51 (.502)
K4. Demonstrate knowledge of epidemiology	.199–.348	DI A3	.83 (.378)	DV K3	.49 (.502)
K5. Understand population health variability	.181–.338	DV K4	.83 (.379)	DII A3	.47 (.501)
**S1. Understand framework to assess communities**	.269–.487	DIII K5	.82 (.380)	DII S3	.47 (.501)
S2. Ask questions to elicit patient preferences	.230–.379	DIII K4	.82 (.380)	DIII K3	.46(.500)
S3. Elicit information in family-centered context	.161–.328	DII A1	.82 (.388)	DII K3	.46 (.500)
**S4. Collaborate with communities to address needs**	.234–.409	DII S2	.81 (.392)	DII A1	.46 (.500)
S5. Recognize institutional cultural issues	.271–.440	DII K4	.81 (.394)	DV S2	.45 (.499)
A1. Exhibit comfort when discussing cultural issues	283–.431	DV K3	.81 (.394)	DIII K5	.42 (.496)
A2. Nonjudgmental listening to health beliefs	.260–.387	DIII S4	.80 (.399)	DV S4	.41 (.493)
A3. Value and address health social determinants	.241–.396	DV A2	.80 (.401)	DV K6	.40 (.492)
A4. Value curiosity, empathy, and respect	.125–.243	DI K1	.79 (.404)	DIII A5	.40 (.491)
DIII. IMPACT OF STEROTYPING AND MEDICAL DECISION-MAKING					
**K1. Describe social cognitive factors**	.327–.497	DV K1	.79 (.407)	DI S1	.40 (.491)
K2. Identify physician bias and stereotyping	.450–.589	DV S2	.79 (.409)	DIII A2	.39 (.489)
K3. Recognize physician own potential for biases	.322–.495	DII A3	.78 (.412)	DV A2	.39 (.489)
K4. Describe the physician-patient power imbalance	.377–.522	DV S3	.78 (.581)	DIII K4	.38 (.486)
K5. Describe community-based elements	.328–.474	DV K5	.78 (.415)	DI K4	.38 (.486)
K6. Describe community partnering strategies	.263–.437	DII K5	.77 (.419)	DIII A4	.37 (.484)
S1. Demonstrate strategies to address/reduce bias	.363–.528	DIII A5	.77 (.424)	DIII S4	.37 (.484)
S2. Describe strategies to reduce physician biases	.384–.548	DV K6	.77 (.424)	DI K3	.36 (.482)
**S3. Show strategies to address bias in others**	.320–.491	DI S1	.76 (.429)	DIV A3	.35 (.478)
S4. Engage in reflection about own beliefs	.359–.509	DIV K4	.76 (.592)	DIII A1	.34 (.475)
S5. Use reflective practices when in patient care	.333–.501	DIII A4	.76 (.430)	DIV K3	.34 (.475)
**S6. Gather and use local data as in HP2010**	.102–.259	DIV A3	.75 (.434)	DIII K2	.33 (.471)
A1. Identify physician biases that affect clinical care	.324–.484	DIII A1	.74 (.436)	DV K1	.32 (.468)
A2. Recognize how physician biases impact care	.372–.515	DV S4	.74 (.437)	DII S5	.32 (.468)
A3. Describe potential ways to address bias	.387–.550	DI A1	.74 (.439)	DIV K4	.29 (.456)
A4. Value the importance of bias on decision-making	.308–.467	DI K3	.74 (.440)	DV K4	.29 (.456)
A5. Value the need to address personal bias	.291–.449	DV S5	.72 (.451)	DI A1	.27 (.446)
DIV. HEALTH DISPARITIES AND FACTORS INFLUENCING HEALTH					
K1. Describe factors that impact health	.182–.312	DIV K3	.71 (.453)	DIV K6	.27 (.446)
K2. Understand social determinants of health	.272–.412	DII S3	.71 (.454)	DV S3	.26 (.442)
K3. Describe systemic & medical encounter issues	.289–.454	DIV K6	.71 (.455)	DIV A2	.26 (.442)
K4. Identify and discuss key areas of disparities	.362–.568	DV K2	.71 (.456)	DIII S5	.26 (.438)
**K5. Describe community-based elements**	.374–.542	DIII A3	.70 (.460)	DI K1	.26 (.438)
K6. Discuss barriers to eliminating health disparities	.356–.519	DIII S2	.69 (.461)	DII K1	.25 (.435)
**S1. Critically appraise literature on disparities**	.293–.465	DIII S1	.69 (.463)	DIII S1	.24 (.430)
**S2. Describe methods to identify community leaders**	.308–.475	DIV A2	.68 (.467)	**DI S3**	.24 (.426)
**S3. Propose a community-based health intervention**	.328–.499	DII S5	.68 (.469)	DV S5	.24 (.426)
**S4. Strategize ways to counteract bias**	.390–.558	DIII S5	.67 (.469)	**DII S4**	.24 (.426)
**A1. Recognize disparities amenable to intervention**	.351–.521	DI K4	.67 (.470)	DV K2	.23 (.422)
**A2. Value the historical impact of racism**	.332–.499	**DIV K5**	.64 (.481)	DIII A3	.23 (.422)
A3. Value eliminating disparities	.322–.482	**DIII K1**	.63 (.482)	DIII S2	.23 (.422)
					
DV. CROSS-CULTURAL CLINICAL SKILLS					
K1. Identify community beliefs & health practices	.397–.548	**DI S2**	.63 (.482)	**DIII K1**	.22 (.417)
K2. Describe cross-cultural communication models	.397–.559	**DIV A1**	.62 (.487)	**DIII K6**	.22 (.412)
K3. Understand physician-patient negotiation	.240–.390	**DIV S4**	.61 (.489)	DV K5	.21 (.408)
K4. Describe the functions of an interpreter	.464–.606	DII K1	.59 (.492)	**DI S2**	.21 (.408)
K5. List effective ways of working w. interpreter	.496–.646	**DI S3**	.58 (.495)	**DIV A1**	.18 (.386)
K6. List ways to enhance patient adherence	.284–.442	**DIII K6**	.56 (.496)	**DIV K5**	.18 (.386)
S1. Elicit a culture, social, and medical history	.236–.379	**DII S4**	.56 (.497)	**DII S1**	.24 (.430)
S2. Use negotiating and problem-solving skills	.260–.414	**DII S1**	.55 (.644)	**DIV S1**	.15 (.361)
S3. Identify and collaborate with interpreter	.416–.618	**DIV S3**	.55 (.498)	**DIV S4**	.13 (.340)
S4. Assess and enhance patient adherence	.253–.414	**DIV S1**	.53 (.499)	**DIV S3**	.13 (.340)
S5. Recognize and manage the impact of bias	.401–.562	**DIII S3**	.53 (.499)	**DIII S3**	.13 (.332)
**A1. Respect patient's cultural beliefs**	.282–.414	DIV S2	.45 (.498)	DIII S6	.11 (.315)
A2. Acknowledge the impact of physician biases	.335–.486	DIII S6	.29 (.455)	DIV S2	.06 (.230)

LEGEND

D = Domain K = Knowledge S = Skill A = Attitude

CI = Confidence Interval for difference in ‘yes’ responses for faculty vs students

*Shaded 19 items used to derive lowest quartile (14 common items in bold) for faculty and student ‘Yes’ responses

**Bold**=Lowest quartile common items for both faculty and students

## Appendix II

**Table 5:** Cronbach's alpha coefficients and corrected item-total score correlation coefficients for 42 items comprising the revised TACCT^a^^,^^b^ (Cronbach alpha for Knowledge=.914, Skill=.923, Attitude=.857 on 42-item TACCT; total 42-item TACCT ICC for students and faculty responses=.905)

**DOMAIN I - Health Disparities** (α=.872)	
**Learning Objectives**	r_i − t_
K-1. Define race, ethnicity and culture (DIK1^c^)	.507
K-2. Identify patterns of national data (D1K3)	.557
K-3. Describe patterns of health disparities (DIIIK5)	.593
K-4. Identify key areas of disparities (DIVK4)	.691
K-5. Discuss barriers to eliminating health disparities (DIVK6)	.690
S-1. Concretize epidemiology of disparities (DIS3)	.546
S-2. Gather and use data 2010 (DIIIS6)	.415
S-3. Critically appraise lit. on disparities (DIVS1)	.590
A-1. Recognize disparities amenable to intervention (DIVA1)	.667
A-2. Value eliminating disparities (DIVA3)	.653
**DOMAIN II Community Strategies** (α=.845)	
**Learning Objectives**	
K-1. Describe challenges in cross-cultural community (DIIK3)	.486
K-2. Understand population health variability (DIIK5)	.475
K-3. Describe community-based elements (DIVK5)	.645
K-4. Identify community beliefs and health practices (DVK1)	.607
S-1. Collaborate with communities (DIIS4)	.608
S-2. Describe methods to identify community leaders (DIVS2)	.605
S-3. Propose a community-based health intervention (DIVS3)	.647
A-1. Value and address social health determinants (DIIA3)	.607
**DOMAIN III - Bias/Stereotyping** (α=.827)	
**Learning Objectives**	
K-1. Identify how race and culture relate to health (DIK2)	.452
K-2. Identify physician bias and stereotyping (DIIIK2)	.577
S-1. Demonstrate strategies to address/reduce bias (DIIIS1)	.701
S-2. Describe strategies to reduce physician bias (DIIIS2)	.713
S-3. Show strategies to reduce bias in others (DIIIS3)	.615
A-1. Value historical impact of racism (DIVA2)	.529
**DOMAIN IV - Communication skills specific to cross-cultural communication** (α=.875)	
**Learning Objectives**	
K-1. Recognize patients’ healing traditions and beliefs (DIIK2)	.542
K-2. Describe cross-cultural communication models (DVK2)	.605
S-1. Discuss race and culture in the medical interview (DIS1)	.531
S-2. Elicit a culture, social and medical history (DVS1)	.660
S-3. Use physician assessment tools (DIS2)	.408
S-4. Elicit information in family-centered context (DIIS3)	.537
S-5. Use negotiating and problem-solving skills (DVS2)	.664
S-6. Assess and enhance adherence (DVS4)	.709
A-1. Respect patient's cultural beliefs (DVA1)	.696
A-2. Nonjudgmental listening to health beliefs (DIIA2)	.610
**DOMAIN V - Use of Interpreters** (α=.857)	
**Learning Objectives**	
K-1. Describe functions of an interpreter (DVK4)	.767
K-2. List effective ways of working with interpreter (DVK5)	.735
S-1. Identify and collaborate with an interpreter (DVS3)	.685
**DOMAIN VI - Self-reflection, culture of medicine** (α=.803)	
**Learning Objectives**	
K-1. Describe the physician-patient power imbalance (DIIIK4)	.526
S-1. Recognize institutional cultural issues (DIISV)	.491
S-2. Engage in reflection about own beliefs (DIIIS4)	.641
S-3. Use reflective practices in patient care (DIIIS5)	.634
A-1. Value the need to address personal bias (DIIIA5)	.648
**Cronbach's α=.964 for the revised 42-item TACCT**	

^a^Because the intraclass correlation coefficient = .905 between medical student and faculty responses on the new TACCT, item analysis statistics were computed using their combined, unweighted response data.

^b^Correlations of each individual item within a domain and the sum score of the domain's items, corrected by removing the contribution of the individual item from the total score.

^c^For original 67-item TACCT domain (D) and knowledge/skill/attitude (K, S, A) learning objectives referenced in parentheses in Table 5, see http://www.aamc.org/meded/tacct/start.htm^12^
